# Supplementary material for: Macrocyclic peptides exhibit antiviral effects against influenza virus HA and prevent pneumonia in animal models
Source: Nat Commun. 2021 May 11;12:2654. doi: 10.1038/s41467-021-22964-w (PMC8113231; doi:10.1038/s41467-021-22964-w)
Supplement: Supplementary file 1 — Supplementary Information [file 41467_2021_22964_MOESM1_ESM.pdf]

Supplementary Information

Macrocyclic peptides exhibit antiviral effects against influenza virus HA and prevent pneumonia in animal models

Makoto Saito[1,*], Yasushi Itoh[2,*], Fumihiko Yasui[1,*], Tsubasa Munakata[1], Daisuke Yamane[1], Makoto Ozawa[3], Risa Ito[4], Takayuki Katoh[4], Hirohito Ishigaki[2], Misako Nakayama[2], Shintaro Shichinohe[2], Kenzaburo Yamaji[1], Naoki Yamamoto[1], Ai Ikejiri[1], Tomoko Honda[1], Takahiro Sanada[1], Yoshihiro Sakoda[5], Hiroshi Kida[6], Le Thi Quynh Mai[7], Yoshihiro Kawaoka[8], Kazumasa Ogasawara[2], Kyoko Tsukiyama-Kohara[3,†], Hiroaki Suga[4,†], and Michinori Kohara[1,†]

[1] Department of Microbiology and Cell Biology, Tokyo Metropolitan Institute of Medical Science, 2-1-6 Kamikitazawa, Setagaya-ku, Tokyo 156-8506, Japan

[2] Division of Pathogenesis and Disease Regulation, Department of Pathology, Shiga University of Medical Science, Setatsukinowa, Otsu, Shiga 520-2192, Japan

[3] Transboundary Animal Diseases Center, Joint Faculty of Veterinary Medicine, Kagoshima University, 1-21-24 Korimoto, Kagoshima 890-0065, Japan

[4] Department of Chemistry, Graduate School of Science, The University of Tokyo, 7-3-1 Hongo, Bunkyo-ku, Tokyo 113-0033, Japan

[5] Laboratory of Microbiology, Faculty of Veterinary Medicine, Hokkaido University, Kita-18 Nishi-9, Sapporo 060-0818, Japan

[6] Hokkaido University Research Center for Zoonosis Control, Kita-18 Nishi-9, Sapporo 060-0818, Japan

[7] National Institute of Hygiene and Epidemiology, Hanoi, Vietnam

[8] Division of Virology, Department of Microbiology and Immunology, The Institute of Medical Science, The University of Tokyo, Tokyo 108-0071, Japan

Supplementary Figures 1-11
Supplementary Tables 1-2

[†]Send correspondence to kohara-mc@igakuken.or.jp

a

Molecule	Antibodies	HA trimer	Macrocycles
MW (Da)	150,000	75,000 x 3	1,200–2,000

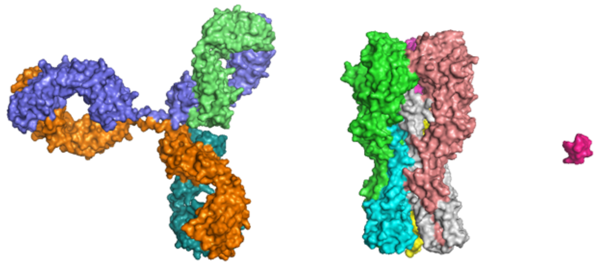

b

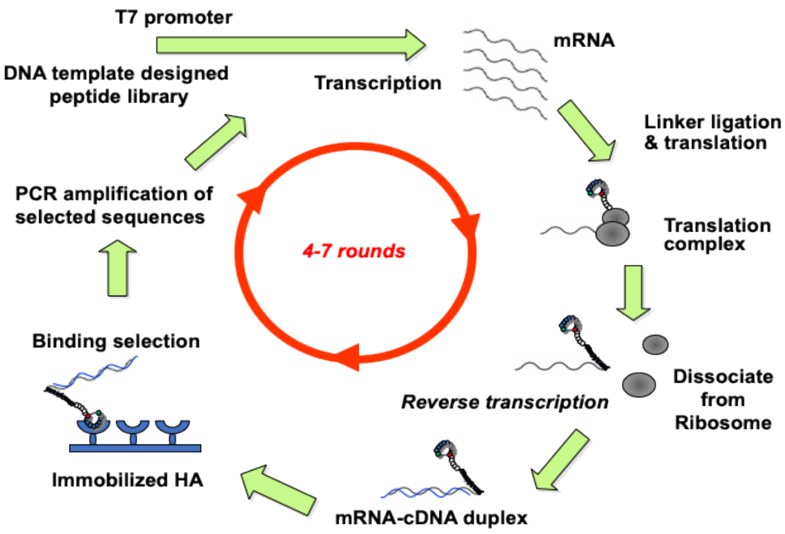

c

iHA	sequence
1	ClAc - WHQWNDLGYLYLPCG- NH_2
2	ClAc - WHQWNDQGYLYLPCG- NH_2
3	ClAc - WHQWNDEGYLYLPCG- NH_2
4	ClAc - WHQWNSEGYLYLDCG- NH_2
5	ClAc - WAQWNDEGYLFLPCG- NH_2
6	ClAc - WHQWNDQGYLYIPCG- NH_2
7	ClAc - WSWLNDEGWGFLPCG- NH_2
8	ClAc - WVRWKPVFAFHPF CG- NH_2
9	ClAc - WRFPVIFRDYLHS CG- NH_2
10	ClAc - WRFPTILPGYRHS CG- NH_2
11	ClAc - WRFKKIFGWAHHA CG- NH_2
12	ClAc - WVIPEWAKLYRFN CG- NH_2
13	ClAc - WPYRARPFPIILT CG- NH_2
14	ClAc - WPYLAGQWPVILW CG- NH_2
15	ClAc - WPSFAGTFPQILI CG- NH_2
16	ClAc - WNIGIPTRWSRIY CG- NH_2
17	ClAc - WWNPITFSSWVWR CG- NH_2
18	ClAc - WQWIANRRYTFEW CG- NH_2
19	ClAc - WGLFSNRRYTFTW CG- NH_2
20	ClAc - WGSFHRGRYAFTW CG- NH_2
21	ClAc - WSGFRERVFWLTW CG- NH_2
22	ClAc - WFVPSPWPKILQP CG- NH_2
23	ClAc - WLIQWCCPYGTAP CG- NH_2
24	ClAc - WWLDPYWLTWYTCG- NH_2
100	ClAc - WTGDFF SSHYTVPR C- NH_2
101	ClAc - WRVS FTY FS YTPS C- NH_2
102	ClAc - WSFGHVHYSV FNAVC- NH_2
103	ClAc - WTGTHVRYTV FNASC- NH_2

Supplementary Figure 1. Screening of iHA macrocycles that inhibit influenza virus replication by binding to HA.

(a), Comparison of molecular sizes of antibody, HA, and macrocycle. Schematic diagrams of antibody (left), HA (middle), and macrocycle (right) are shown. (b), Workflow for library screening of macrocyclic peptides that bind to influenza viral HA. The library of cDNAs encoding random amino acid sequences was transcribed, and then mRNAs were translated in a cell-free translation system including tRNAs with replacement of the initiation codon. In this system, chloro-acetylated tryptophan was used as the amino acid corresponding to the AUG initiation codon instead of methionine, which formed a non-reducible thioether bond with the C-terminal cysteine, resulting in the formation of a cyclic structure. A library of mRNA-linked macrocyclic peptides was generated with flexizyme-based in vitro translation and screened with a recombinant baculovirus expressing HA protein derived from the highly pathogenic avian influenza virus A/Vietnam/1203/04 (H5N1; clade 1) or the recombinant vaccinia virus expressing HA protein derived from the highly pathogenic avian influenza virus A/Bar-Headed goose/Qinghai Lake/1A/05 (H5N1; clade 2.2). The mRNAs associated with the HA-binding peptides were converted to cDNAs with RT-PCR and subjected to the next round of affinity selection. The initial library size was about 4 × 10E13. Through five rounds of selection, we found an appreciable increase in the cDNA recovery efficiency. (c), Amino acid sequences of the selected HA-binding macrocycle candidates. Sequencing of 69 molecular clones from the selected cDNAs revealed 28 candidates for inhibitors of HA (iHA). The macrocyclic structure (thioether bond) is formed between chloro-acetylated tryptophan (amino acids are represented by green characters) and the C-terminal cysteine (represented by red characters). The purple characters indicate the N-methylamino acids. The clones that exhibited moderate and the highest inhibition of virus plaque formation are highlighted in blue and yellow, respectively. Source data are provided as a Source Data file.

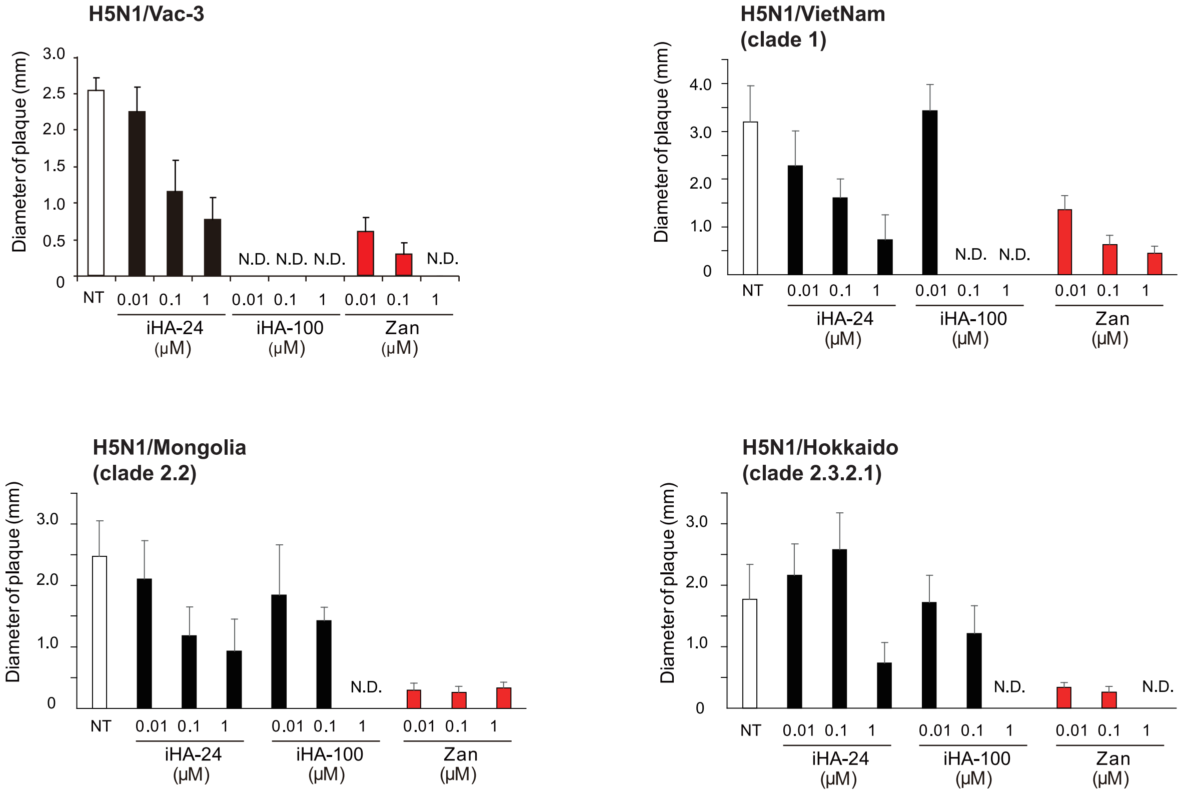

Supplementary Figure 2. Both iHA-24 and iHA-100 reduced the size of viral plaques. The diameters (error bars indicate SD of 10 randomly selected plaques) of the plaques formed under agarose medium in the absence (None) or presence of iHA-24, iHA-100 (0.01, 0.1, or 1 μM each), or zanamivir (1 μM) are shown. Results are shown as mean values and are representative of three biologically independent experiments. Vertical bars indicate SD of three replicates. N.D., not detected. Source data are provided as a Source Data file.

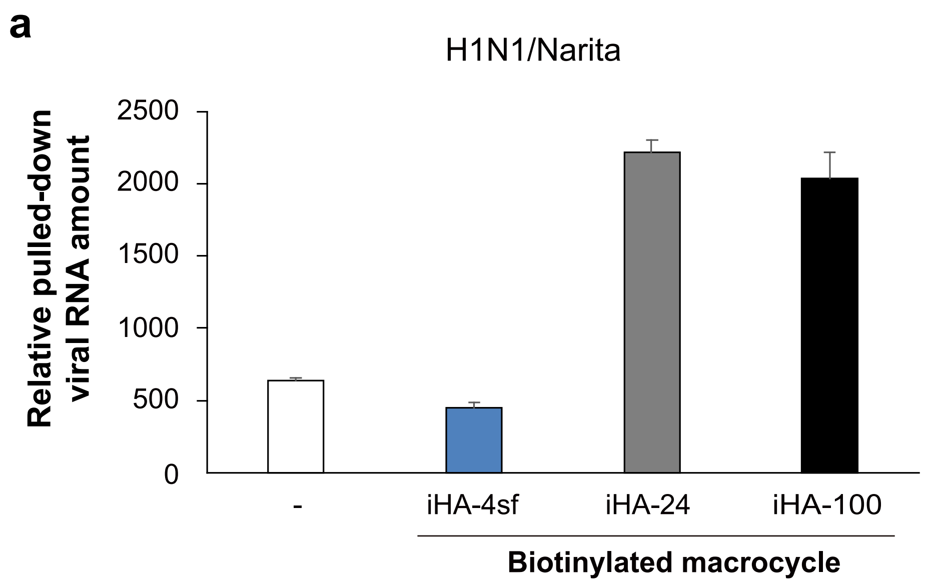

Supplementary Figure 3. Binding of iHA-24 and iHA-100 to H1N1 and H2N2 virions. RNAs were extracted from H1N1/Narita (a) and H2N2/Adachi (b) bound to biotinylated iHA-100 and subjected to real-time PCR to quantify viral RNAs for the M gene. Error bars indicate the SD of three replicates. Results are representative of two biologically independent experiments. Source data are provided as a Source Data file.

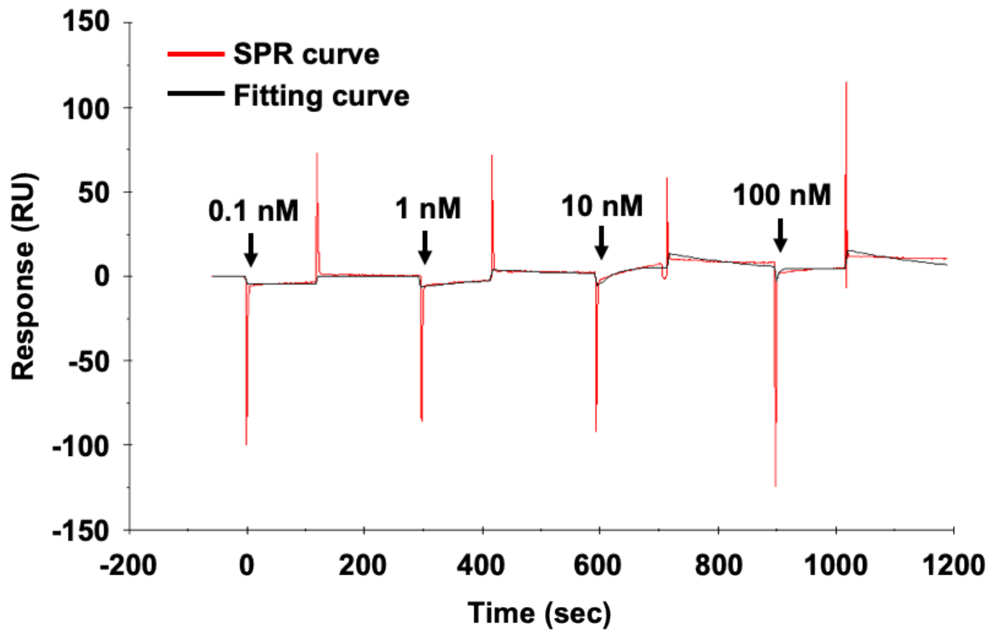

Supplementary Figure 4. Single-cycle kinetic analysis of the interaction between HA and iHA-100 by surface plasmon resonance (SPR).

SPR sensorgrams for iHA-100 binding to H5 HA are shown. Single-cycle kinetics were run on a Biacore X100 Plus Package to investigate the binding kinetics of iHA-100 against influenza A virus HA. Experiments were performed in triplicate and representative data are shown. Source data are provided as a Source Data file.

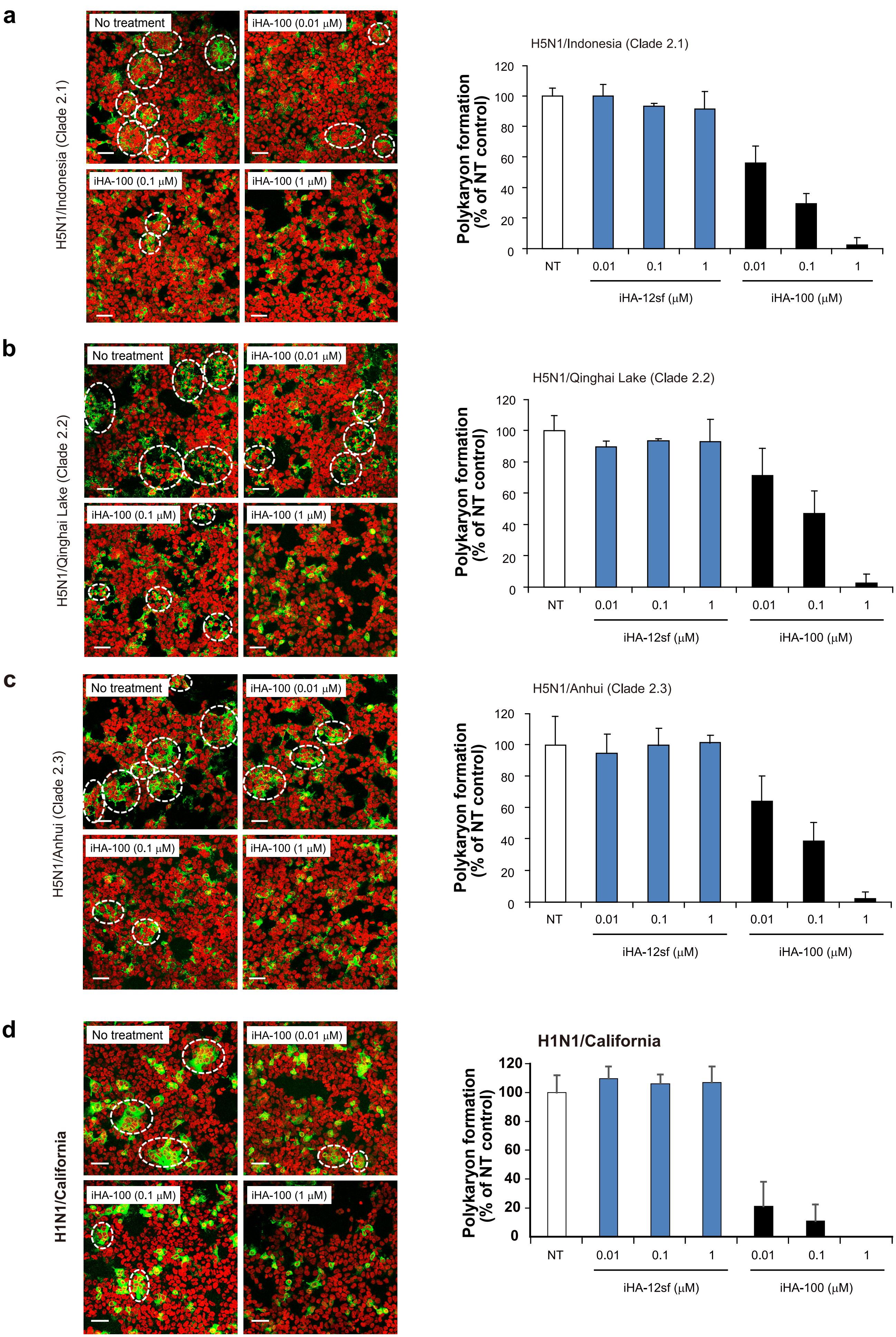
a
No treatment
iHA-100 (0.01 μM)
iHA-100 (0.1 μM)
iHA-100 (1 μM)
H5N1/Indonesia (Clade 2.1)
Polykaryon formation (% of NT control)
NT
0.01
0.1
1
iHA-12sf (μM)
iHA-100 (μM)
b
H5N1/Qinghai Lake (Clade 2.2)
c
H5N1/Anhui (Clade 2.3)
d
H1N1/California

Supplementary Figure 5. Effect of iHA-100 on H5 and H1 HA-mediated polykaryon formation.

(a), HEK293 cells transiently expressing HA from H5N1/Indonesia (clade 2.1), (b), H5N1/Qinghai Lake (clade 2.2), (c), H5N1/Anhui (clade 2.3), and (d), A/California/07/2009 (H1N1) were trypsinized and exposed to low pH. After incubation at 37°C for 6 h, HA (green in left images) and nuclei (red in left images) were visualized with immunofluorescence staining, and the polykaryon formation efficiency was quantified. Right panel: Percentage (%) of polykaryon formation normalized to the no treatment (NT) control is indicated. Error bars indicate SD of 10 randomly selected fields. Results are shown as mean values and are representative of three biologically independent experiments. Source data are provided as a Source Data file.

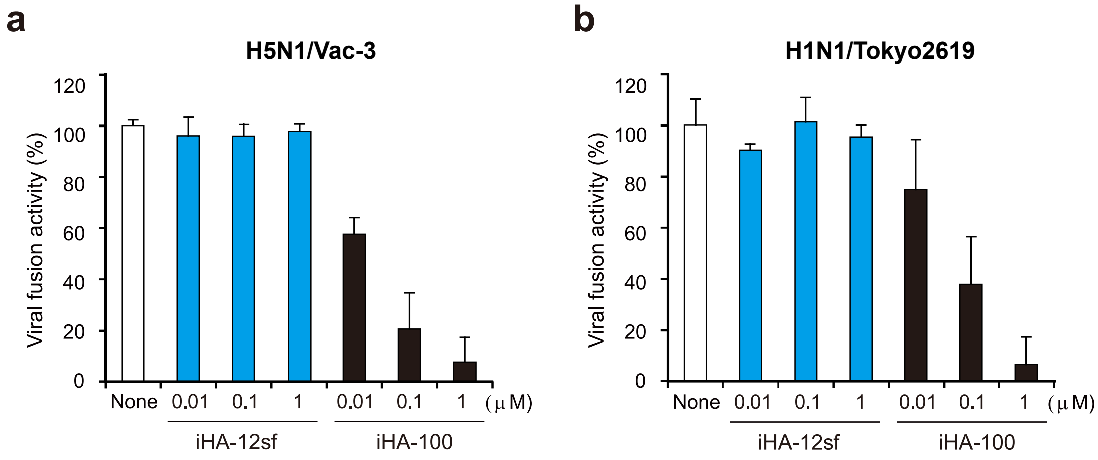

Supplementary Figure 6. Effect of iHA-100 on influenza virus-mediated red blood cell (RBC) fusion.

Chicken RBCs mixed with H5N1/Vac-3 (a) or H1N1/Tokyo2619 (b) and macrocyclic peptide iHA-100 or -12sf (0.01, 0.1, or 1 μM each) were exposed to low pH. After incubation at 37°C for 60 min, the NADPH concentrations in the clarified supernatants were determined by measuring the optical density at 340 nm. Both H5N1/Vac-3 and H1N1/Tokyo2619 virus-mediated RBC membrane fusion was inhibited by iHA-100 in a dose-dependent manner, whereas iHA-12sf even at 1 μM did not reduce RBC membrane fusion. iHA-12sf is a control macrocycle, which consists of a scrambled iHA-12 amino acid sequence. Results are shown as mean values and are representative of two independent experiments. Error bars indicate the SD of three replicates. Source data are provided as a Source Data file.

a **HA escape mutations against iHA-100**

H3 numbering / subtype/strain	HA1 318	HA2 10 (339)	HA2 46 (375)	HA2 49 (378)	HA2 110 (439)	HA2 136 (465)
**H1N1/PR8**	T	I → V	N → D	T	F → S	G → R
**H5N1/Vac-3**	T → K	I	D	T → N	F	G

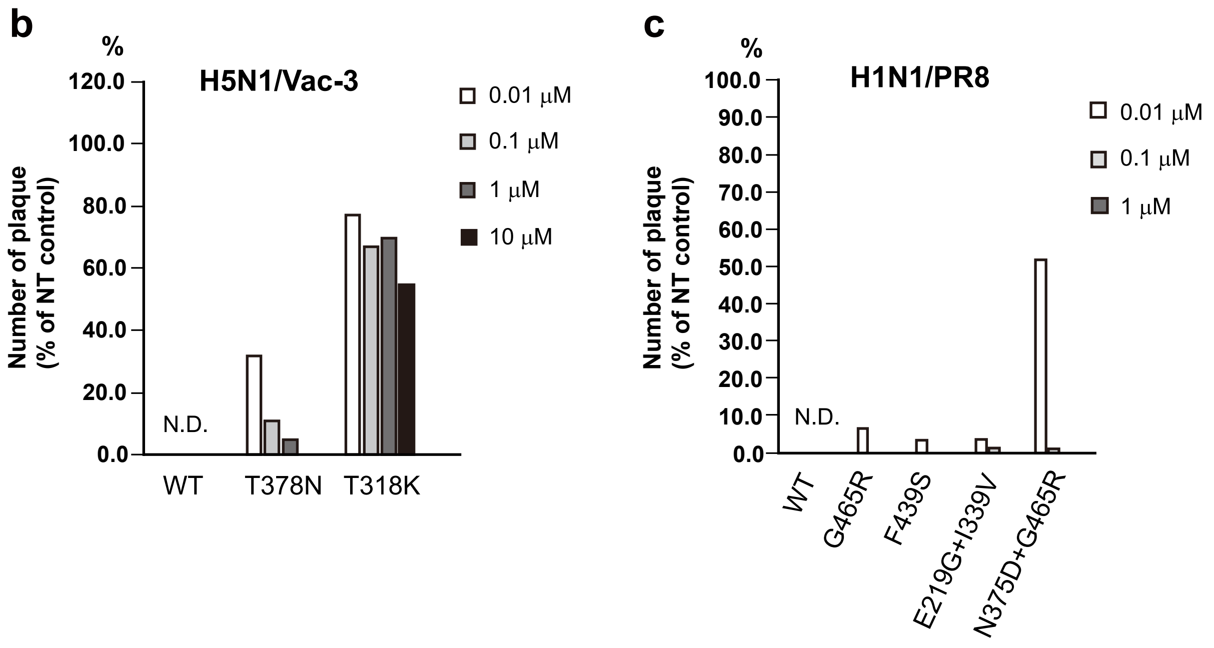

Supplementary Figure 7. Structure of HA and escape mutants.
(a), HA escape mutations against iHA-100. Light-green characters: hydrophobic amino acids. Light-blue characters: polar amino acids. Purple characters: acidic amino acids. Orange characters: basic amino acids. b and c, The resistance of escape mutants against iHA-100 treatment. MDCK cells infected with 100 PFU of H5N1/Vac-3-derived escape mutants (b) or H1N1/PR8-derived escape mutants (c) that were treated with either iHA-100 (0.01, 0.1, 1, or 10 µM for H5N1/Vac-3 and 0.01, 0.1 or 1 µM for H1N1/PR8) or DMSO (vehicle) for inhibition of cell attachment. At 1 h post-infection, cells were overlaid with agarose medium containing iHA-100 (0.01, 0.1, 1, or 10 µM for H5N1/Vac-3 and 0.01, 0.1 or 1 µM for H1N1/PR8) or DMSO for inhibition of the fusion step. After 48-72 h of incubation, the percent plaque reduction compared to the untreated control was calculated. Results are shown as mean values and are representative of two biologically independent experiments. N.D., not detected. Source Data are provided as a Source Data file.

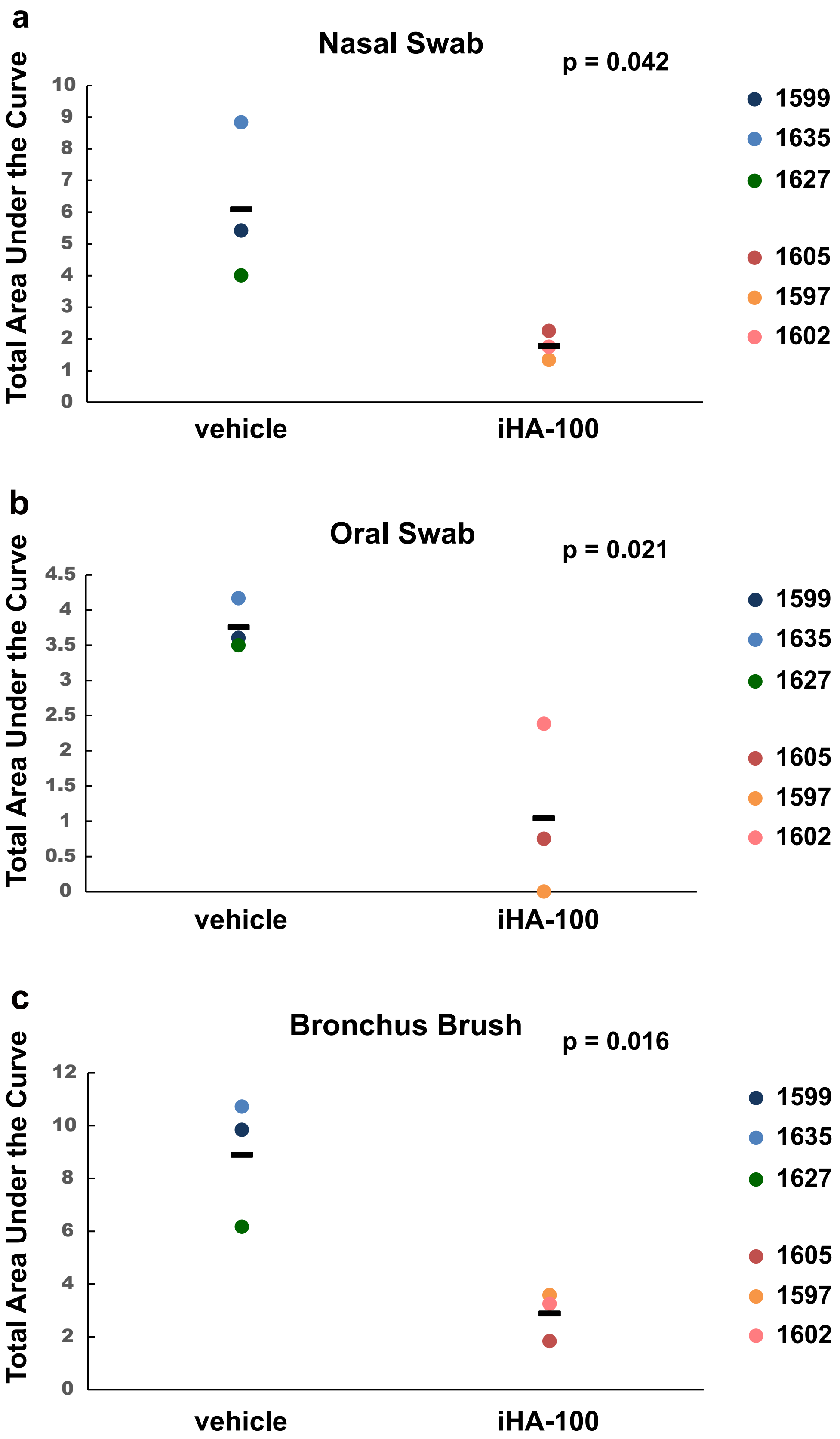
a
Nasal Swab
p = 0.042
Total Area Under the Curve
10
9
8
7
6
5
4
3
2
1
0
vehicle
iHA-100
1599
1635
1627
1605
1597
1602
b
Oral Swab
p = 0.021
Total Area Under the Curve
4.5
4
3.5
3
2.5
2
1.5
1
0.5
0
vehicle
iHA-100
1599
1635
1627
1605
1597
1602
c
Bronchus Brush
p = 0.016
Total Area Under the Curve
12
10
8
6
4
2
0
vehicle
iHA-100
1599
1635
1627
1605
1597
1602

Supplementary Figure 8. Summations of virus titer levels after treatment (day 3 to day 7). The accumulated virus shedding after treatment (3-7 dpi) in (a), nasal swabs, (b), oral swabs, and (c), bronchus swabs shown in Fig. 4f-h were calculated as the area under the curve. P values were calculated by Student's t test (two-sided, unpaired). Source data are provided as a Source Data file.

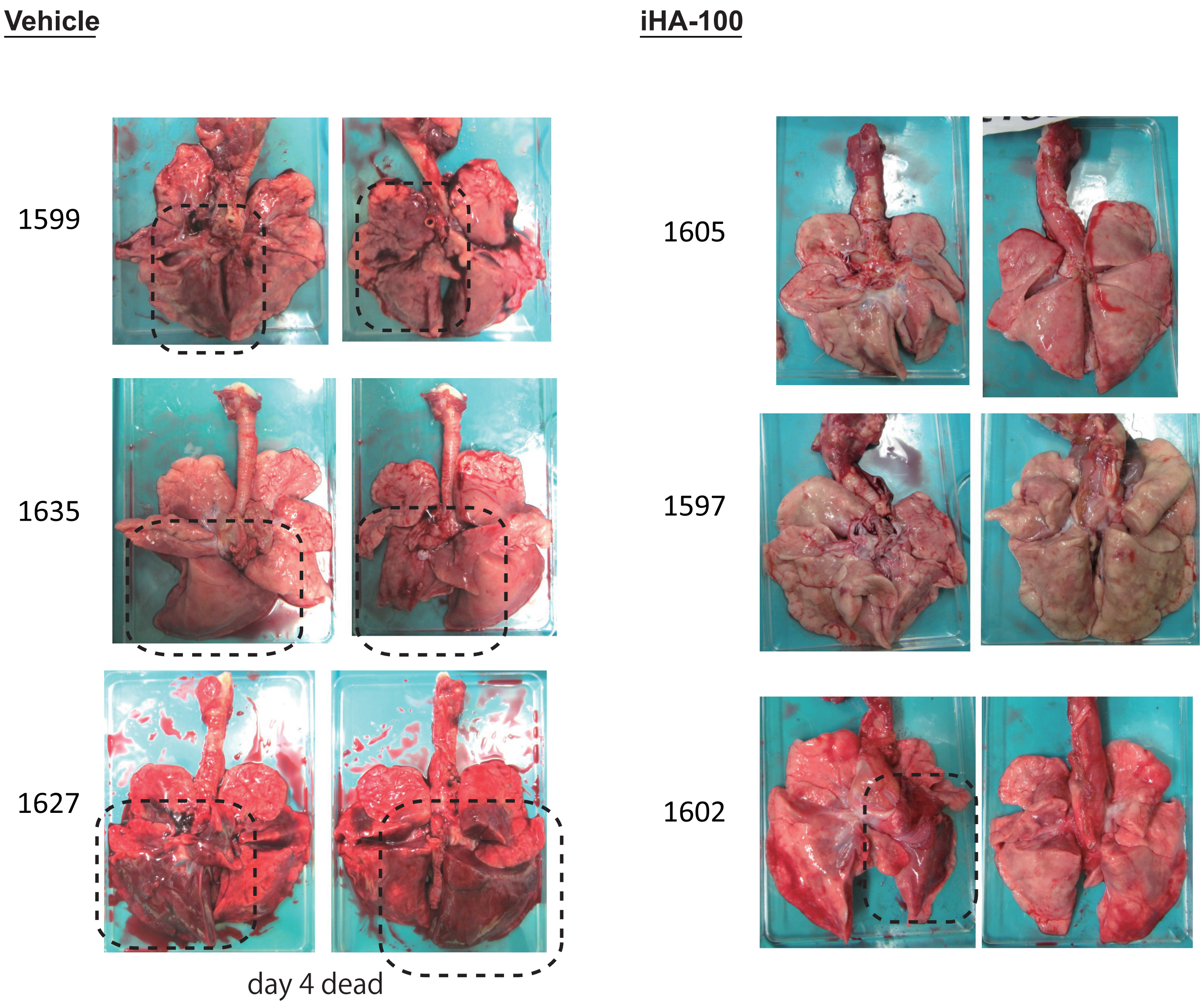

Supplementary Figure 9. Macroscopic findings of the extirpated lungs.
The lungs of the vehicle-treated monkeys exhibited obvious inflammatory lesions (dashed boxes) compared with the lungs of the iHA-100-treated monkeys. Source data are provided as a Source Data file.

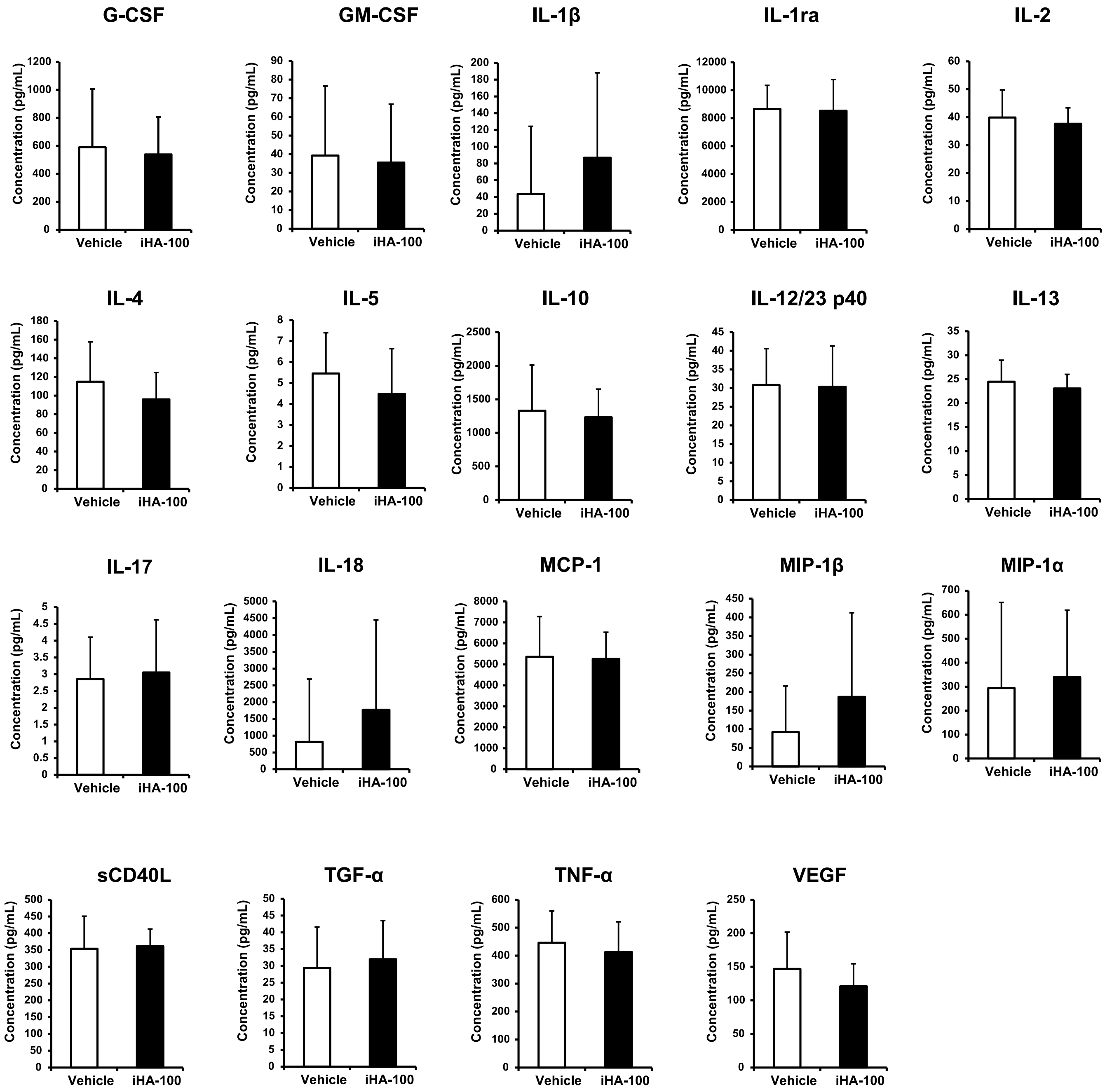

Supplementary Figure 10. Comprehensive cytokine analysis of lungs extirpated from H5N1-infected monkeys.

Each lobe of the lung was homogenized and used for the multiplex assay for 23-plex non-human primate cytokines. Data from lung six-lobe (RU, RM, RL, LU, LM, and LL) homogenates of each monkey were measured. Results of 19 cytokines other than IL-6, IFN-g, and IL-15 are shown. Results are shown as mean values and are representative of two biologically independent experiments. Vertical bars indicate SD of three monkeys. Source data are provided as a Source Data file.

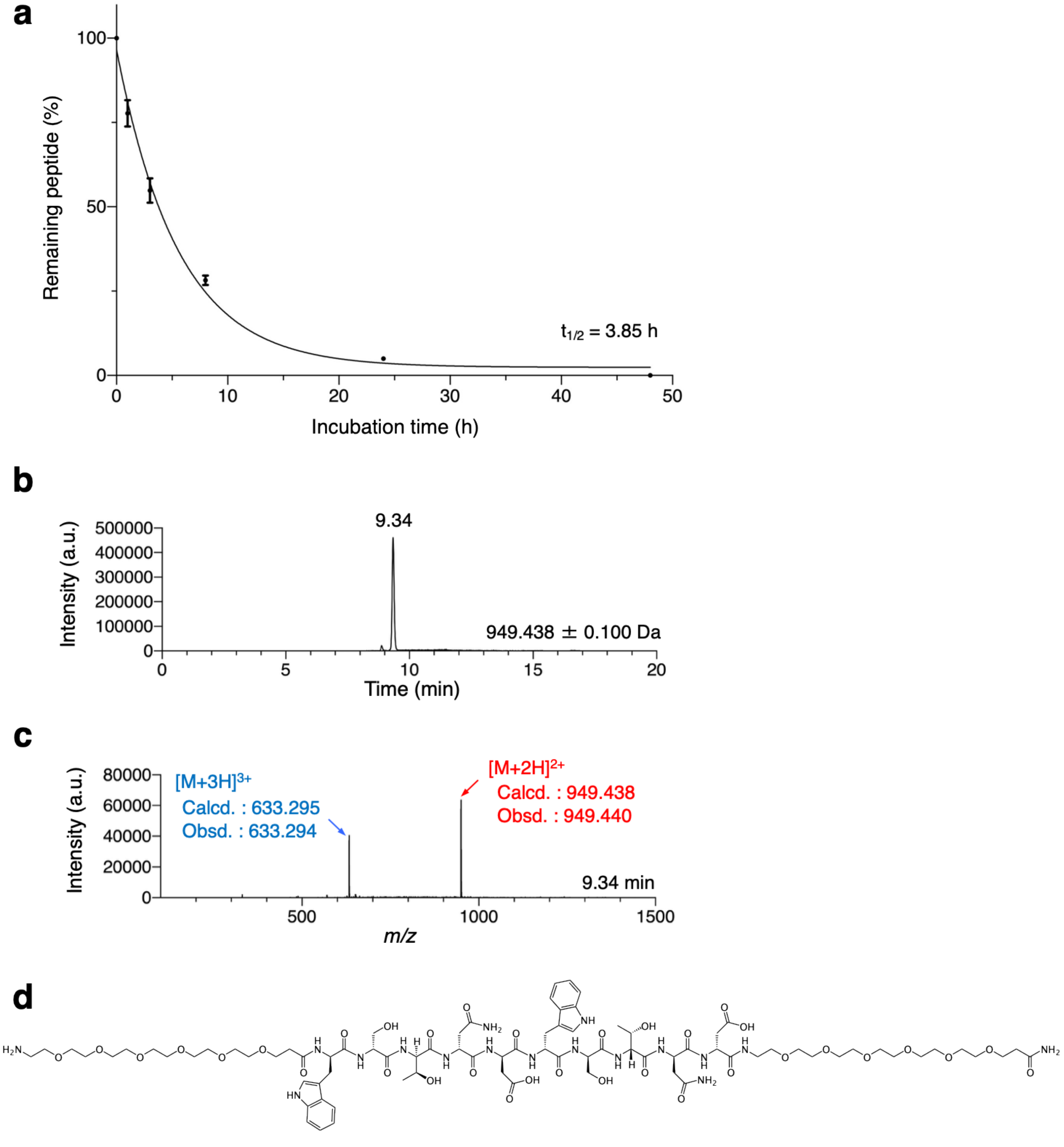

Supplementary Figure 11. Stability of the iHA-100 peptide in serum.
(a) iHA-100 was co-incubated with an internal standard peptide in human serum at 37°C. The relative intensity of iHA-100 to the standard peptide was analyzed with LC/MS at 0, 1, 3, 8, 24, and 48 h. The relative intensity at 0 h was defined as 100% and is indicated as a mean value. Error bars indicate SD of three replicates. (b, c) Mass chromatogram (b) and mass spectrum (c) of the reaction mixture after 3 h of incubation. Red and blue arrows indicate [M+2H]2+ and [M+3H]3+ ions, respectively. Calcd. and Obsd. stand for calculated and observed m/z values. (d) Chemical structure of the internal control peptide used in the serum stability assay. Source data are provided as a Source Data file.

**Supplementary Table 1**				
**EC50 (μM)**				
Figure	influenza virus	iHA-24	iHA-100	Zanami
Fig. 1 d	H5N1/Vac-3	0.036	<0.01	0.042
Fig. 1 e	H5N1/Vietnam	0.082	<0.01	NT
Fig. 1 f	H5N1/Mongolia	0.702	<0.01	<0.01
Fig. 1 g	H5N1/Hokkaido	0.107	<0.01	<0.01
Fig. 1 h	H1N1/PR8	<0.01	<0.01	0.115
Fig. 1 i	H1N1/Tokyo2619	<0.01	<0.01	>1
Fig. 1 j	H1N1/Narita	NT	<0.01	>1
Fig. 1 k	H2N2/Adachi	2.46	<0.01	0.06
			NT: not tested	

Supplementary Table 1. Effect of the selected macrocycles on plaque formation by Group 1 influenza viruses.
Among these macrocycles, iHA-24 and iHA-100 remarkably reduced the plaque numbers.

Gene name	Forward (5' to 3')	application
A-MP-F	CTT CTA ACC GAG GTC GAA ACG TA	Influenza virus A, Matrix gene
A-MP-R	TTG GAC AAA GCG TCT ACG CTG C	Influenza virus A, Matrix gene
B-MP-F	CAG GGC TCA TAG CAG AGC	Influenza virus B, Matrix gene
B-MP-R	AAG AGA TCT CAG CAC TCC AAT GTT GC	Influenza virus B, Matrix gene
SO-HA-F	GAG CTA AGA GAG CAA TTG A	S-OIV HA gene
SO-HA-R	TAG CAC GAG GAC TTC TTT CC	S-OIV HA gene
H5-F	ACA TGC CCA AGA CAT ACT GGA AAA GAC ACA CAA CGG	H5 HA
H5-R	ATG TAA GAC CAT TCC GGC ACA TTG ATG A	H5 HA

Supplementary Table 2. Primer sequences used in this study.
Viral RNAs from precipitated viruses were quantified for the M gene with the real-time thermal cycler CFX96 and using primers listed in Supplementary Table 2.
